# Multi-omic single cell sequencing: Overview and opportunities for kidney disease therapeutic development

**DOI:** 10.3389/fmolb.2023.1176856

**Published:** 2023-04-05

**Authors:** Steven Pregizer, Thom Vreven, Mohit Mathur, Luke N. Robinson

**Affiliations:** Visterra Inc., Waltham, MA, United States

**Keywords:** single-cell, multi-omic, scRNA-seq, kidney, integration

## Abstract

Single cell sequencing technologies have rapidly advanced in the last decade and are increasingly applied to gain unprecedented insights by deconstructing complex biology to its fundamental unit, the individual cell. First developed for measurement of gene expression, single cell sequencing approaches have evolved to allow simultaneous profiling of multiple additional features, including chromatin accessibility within the nucleus and protein expression at the cell surface. These multi-omic approaches can now further be applied to cells *in situ*, capturing the spatial context within which their biology occurs. To extract insights from these complex datasets, new computational tools have facilitated the integration of information across different data types and the use of machine learning approaches. Here, we summarize current experimental and computational methods for generation and integration of single cell multi-omic datasets. We focus on opportunities for multi-omic single cell sequencing to augment therapeutic development for kidney disease, including applications for biomarkers, disease stratification and target identification.

## 1 Single cell multi-omic assays

### 1.1 Single cell assays

**FIGURE 1:**
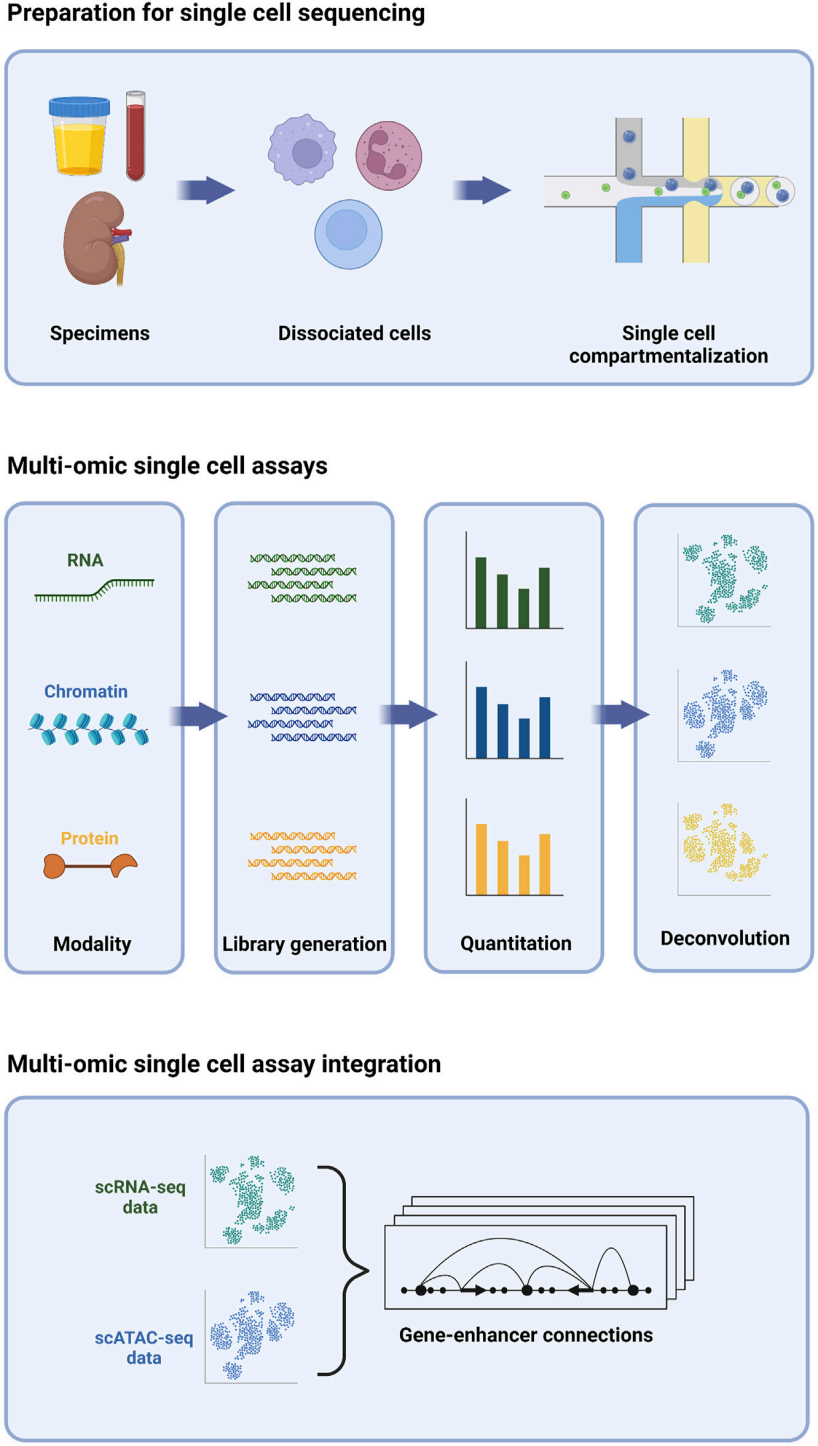
Multi-omic single cell sequencing provides an integrated view of cell biology from heterogeneous tissue sources.

The development of single cell sequencing technology focused initially on profiling the transcriptome (Tang et al., 2009); however, the number of assays that can be adapted to sequencing at the single cell level has undergone rapid growth. Importantly, multiple assays, each measuring a different molecular property, can be performed concurrently on the same cells, enabling a multi-omic deconstruction of individual cells (Figure 1). Collectively, these assays have become a powerful new toolkit for probing myriad aspects of single cells, including transcriptomic, epigenomic, and proteomic signatures. In this review, we will first focus on three different single cell assays.

Single cell transcriptome profiling by RNA-sequencing (scRNA-seq) entails the generation of a barcoded complementary DNA (cDNA) library from the total pool of RNA transcripts present in a given cell at a given time. The chemistries commonly employed for high-throughput single cell cDNA generation typically bias the assay towards the 5’ or 3’ end of the most abundant polyadenylated transcripts (Klein et al., 2015; Macosko et al., 2015; Cao et al., 2017; Zheng et al., 2017), while lower-throughput chemistries offer higher sensitivity and full-length coverage, including non-polyadenylated species (Hagemann-Jensen et al., 2020; Hahaut et al., 2022; Salmen et al., 2022). Regardless of the chemistry used, the transcriptome of each cell is effectively sampled *via* this assay, enabling interrogation of gene expression without the need to pre-select a set of target genes. A key benefit of this target-agnostic approach is the ability to explore fundamental aspects of cell biology without the constraints of *a priori* assumptions. For example, each cell’s “transcriptomic signature” can be used for cell type classification, typically *via* comparison with reference maps or by clustering and manual inspection of expression of marker genes. With unbiased single-cell resolution, novel cell types may be appreciated that were missed by conventional classification schemes, typically relying on a handful of pre-defined markers (Regev et al., 2017). Likewise, changes in gene expression can be deconstructed to understand biological activities and activated pathways in cells, enabling insights to the functional roles and developmental trajectories of different cells that may elude more biased approaches.

Single cell epigenomic profiling by assay for transposase-accessible chromatin (scATAC) entails fragmentation and tagging of the genome with barcoded adapters for high-throughput sequencing by Tn5 transposase (Buenrostro et al., 2015). Because the enzyme preferentially integrates DNA tags into nucleosome-free regions of “open” chromatin, these regions are enriched in scATAC-seq libraries, while regions of “closed” chromatin are not. The enriched regions typically correspond to active regulatory elements; namely, promoters and enhancers. Thus, scATAC-seq provides genome-wide high-resolution maps of regulatory activity on a cell-by-cell basis. These regulatory maps are essential for pinpointing cell type-specific enhancers that elude identification by bulk methods (Buenrostro et al., 2015; Buenrostro et al., 2018). Transcriptional activity is typically presaged by chromatin changes within a given locus (Ma et al., 2020); thus, single cell regulatory maps derived from scATAC-seq are complementary to transcriptomic profiles derived from scRNA-seq and can likewise be used to deconvolute cell types and states, and in some cases, more effectively (Cusanovich et al., 2018; Shema et al., 2019). Finally, the repertoire of active regulatory elements in a given cell type can be interrogated at the primary sequence level to identify over-represented transcription factor binding motifs (Cusanovich et al., 2018)). These molecular “footprints,” in turn, nominate master regulators of transcriptional activity within each cell type. Thus, scATAC-seq offers a bias-free approach for identifying upstream factors governing cell fate and function.

Single cell proteomic profiling by sequencing entails labeling cells with DNA-barcoded antibodies against protein targets, enabling quantitative measurement of their expression. Targets profiled can include those on the cell surface (e.g., CITE-seq, REAP-seq) (Peterson et al., 2017; Shahi et al., 2017; Stoeckius et al., 2017; O'Huallachain et al., 2020; Hwang et al., 2021; Sheng et al., 2022), as well as those within the cytosolic (van Buggenum et al., 2018; Gerlach et al., 2019) and nuclear (Chung et al., 2021) compartments. Sequencing-based single cell proteomic profiling is orthogonal to flow or mass cytometry, which use fluorophore or isotope-coupled antibodies, respectively, to probe antigens on a single-cell level. In contrast to these methods, sequencing-based proteomic profiling allows for as many as 10-fold more proteins to be profiled simultaneously, with studies demonstrating >100 proteins profiled (Su et al., 2020; Nettersheim et al., 2022). The number of proteins profiled, in practice, for sequencing-based single cell proteomic profiling is limited by the availability and inclusion of high-quality antibodies, and thus contrasts somewhat from the assays discussed previously. Nevertheless, sequencing-based single-cell proteomic profiling, even with a limited number of targets, is quite capable of resolving cell types and states (Shahi et al., 2017). Many cell types, especially immune cells, are defined by the proteins they express on their surface, and thus proteomic profiling by sequencing enables more direct relating of data to classic immunophenotyping, e.g., flow cytometry. Finally, sequencing-based single cell proteomic profiling has the capacity to detect post-translational modifications, allowing signaling events to be directly observed, rather than inferred from expression of pathway constituent and/or target genes (van Buggenum et al., 2018; Gerlach et al., 2019).

### 1.2 Integration of multiple single cell assay formats

The conversion of transcript expression, protein expression, and chromatin accessibility to a common sequence-based readout makes it possible to simultaneously obtain multiple modalities from individual cells in a single experiment. The first reports of multi-omic single cell profiling involved the combined profiling of transcriptomes and proteins. These early multi-omic assays included Cellular Indexing of Transcriptomes and Epitopes by Sequencing (CITE-seq) (Stoeckius et al., 2017), and RNA expression and Protein Sequencing (REAP-seq) (Peterson et al., 2017). Several additional variations on this theme have since emerged (Peterson et al., 2017; Gerlach et al., 2019; Chung et al., 2021; Hwang et al., 2021). Joint profiling of protein and transcript expression in the same cells effectively increases the overall amount of information available for unsupervised clustering/neighborhood mapping, thus providing enhanced ability to resolve cell types and states (Stoeckius et al., 2017). Additionally, it allows functional inferences to be drawn between expression of nuclear proteins and the potential impact on gene expression (Chung et al., 2021).

Joint profiling of transcript expression and chromatin accessibility was described shortly after the introduction of CITE-seq and REAP-seq (Cao et al., 2018), and this was likewise followed by several variations (Chen et al., 2019; Zhu et al., 2019; Ma et al., 2020). As with paired protein and transcript expression, the integration of paired chromatin accessibility and transcript expression from single cells can enhance resolution and identification of cell types and states. Importantly, the ability to directly link the regulatory landscape of a single cell with its transcriptome has powerful implications for deconvoluting gene regulatory networks. Changes in a gene’s transcriptional activity can be directly correlated with changes in accessibility of specific regions in the surrounding locus, allowing assignment of regulatory elements to their cognate target genes to be made with much greater accuracy. This, in turn, leads to more robust hypotheses about how common disease-associated variants mapping to such regulatory elements may be exerting their effects (Ma et al., 2020; Kartha et al., 2022).

Joint single cell profiling of chromatin accessibility and protein expression has been described more recently (Mimitou et al., 2021; Swanson et al., 2021). These methods were developed in connection with methods that additionally profile transcriptomes from single cells (Mimitou et al., 2021; Swanson et al., 2021). Together with a similar, recently developed trimodal single cell assay (Chen et al., 2022), these methods herald a sea change in our approach to cell biology. These trimodal methods make it possible, for the first time, to directly observe how changes within the nucleus propagate to the cell surface, and *vice versa*. These methods are still in their infancy, and further refinements will be necessary to aid their widespread adoption; however, they offer a glimpse of what promises to be a more holistic era in cell biology.

### 1.3 Opportunities and challenges for single cell assays in kidney disease

Kidney is an inherently challenging organ for single cell analysis, owing to the need to first dissociate cells from the matrix in which they are embedded. This typically entails enzymatic digestion and/or mechanical disruption, processes that can result in cell death and/or stress responses. The latter can be particularly confounding for single cell analysis, as transcriptional responses to stress may obscure native cellular phenotypes (Adam et al., 2017; O'Flanagan et al., 2019). Use of cold-activated proteases can alleviate this somewhat; however, the greater challenge in sequencing single cells from the kidney is that some cell types are more refractory than others to enzymatic dissociation (Wu et al., 2018). When these refractory cell types are also rare, they can be severely under-represented in single cell datasets. Glomerular cells, including podocytes, mesangial cells, and epithelial cells are a classic example of this conundrum (Wu et al., 2019). This can be overcome to some degree by scaling up the number of cells profiled and adapting the method to target a particular cell type (Chung et al., 2020); however, these approaches are not always feasible, especially with clinical samples.

Single nucleus RNA sequencing (snRNA-seq) has emerged as a helpful alternative to single cell sequencing in cases where cell dissociation is necessary and poses a challenge (Grindberg et al., 2013; Habib et al., 2016; Krishnaswami et al., 2016; Lacarr et al., 2016; Lake et al., 2016; Habib et al., 2017; Martin et al., 2023). Nuclei are more resistant to lysis than cells and contain abundant pre-spliced mRNA, in addition to housing the genomic material. Thus, harsher mechanical disruption techniques can be applied to recover nuclei from difficult tissues without compromising the integrity of their contents. As with scRNA-seq, snRNA-seq can be adapted to profile chromatin accessibility in parallel (Cao et al., 2018; Chen et al., 2019; Zhu et al., 2019; Ma et al., 2020); however, profiling surface protein expression is precluded with this approach. At least one study has shown that snRNA-seq is superior to scRNA-seq for recovery of glomerular cells (Wu et al., 2019). An additional advantage of single nucleus sequencing is its applicability to frozen tissues, thus enabling information to be extracted at single-cell resolution from frozen/archived clinical samples that would otherwise not be amenable to single cell sequencing (Lake et al., 2019; Rousselle et al., 2022).

## 2 Computational approaches for integrated analyses of multi-omic single cell datasets

### 2.1 Overview of integration approaches

The availability of multiple assays, or modalities, for single cell sequencing can provide a much deeper understanding of the biological processes compared to single modalities. On their own, individual modalities can present complementary evidence or serve as independent validation of biological findings. To unlock a greater potential of multi-omic datasets, the modalities need to be explicitly integrated, which can then substantially expand the insights that can be obtained from the individual modalities.

A range of integration approaches and tools have been developed for multi-omic single cell datasets. Some of these were developed for specific combinations of modalities, while others can handle a broad range of modality combinations. Here we outline the key distinguishing features of these tools noting that the selection of an optimal tool is largely determined by the nature of the data and the biological insights that are sought.

Data from multiple modalities can be from the same cells (paired data) or from different but similar cells (unpaired data) (Argelaguet et al., 2021). Paired data provides a direct mapping between the modalities at the cell-to-cell level, and thus allow the most direct integration. Obtaining paired data, however, may not be straightforward or even possible for all combinations of modalities. Additionally, datasets from altogether different origins may need to be combined, such as scATAC-seq and scRNA-seq of PBMCs from different laboratories on different dates. Consequently, there is a need for integration approaches that can handle both paired and unpaired datasets, and computational methods have been developed for handling either situation.

**FIGURE 2:**
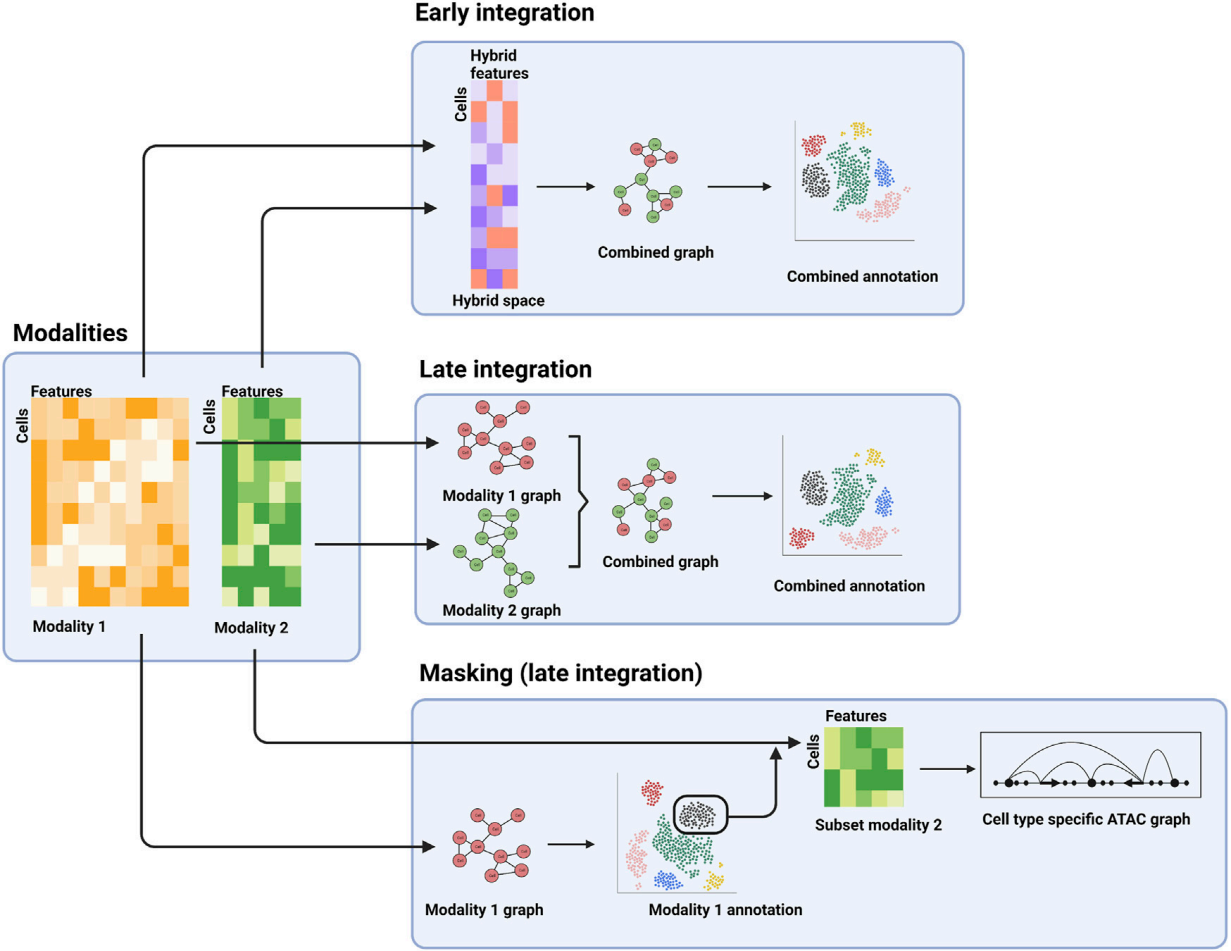
Examples of different stages of integration. The separate modalities (left panel) can be combined into a hybrid space for downstream analysis (top panel), processed separately and combined further downstream (middle-right panel), or the first modality can be used for a cell type-specific analysis of a second modality.

Another principal distinction between various integration methods is the analysis stage when integration is performed: early stage and late stage (Miao et al., 2021a). For early-stage integration methods, the different modality datasets are integrated at the beginning of the analyses (Figure 2). Effectively this creates a new, hybrid modality dataset that can then be used for downstream analysis, thus enabling application of other analysis tools commonly applied to unimodal data. Whereas single cell data is typically high dimensional (e.g., each gene in a scRNA-seq dataset is represented by one dimension), the hybrid dataset is low dimensional. For example, the TotalVI method for integrating transcriptomic and protein data uses a neural network to place the combined data in a 20-dimensional reduced space (Gayoso et al., 2021). Downstream analysis, such as differential expression analysis, can be performed in this space. The generative component of the neural network can be used to relate the results in the reduced dimensionality space back to the original RNA and protein identities. These early-stage approaches lend themselves well to integration of unpaired assays as the hybrid space can accommodate data from either assay type (Miao et al., 2021b).

In contrast, late-stage integration does not explicitly combine the different modality datasets, but rather combines analysis results obtained for the individual modalities. For example, a typical step in single cell analysis is the construction of a neighbor-graph that describes the similarities of the cells. The neighbor-graph is then used as input for clustering analysis or reduced dimensionality visualization, such as a uniform manifold approximation and projection (UMAP) or t-distributed stochastic neighbor embedding (t-SNE) map. In a multi-modal context, neighbor-graphs can be constructed for the individual assays, which can then be merged and subsequently used for integrated clustering analysis, visualization, and possibly cell type and state annotation (Figure 2) (Hao et al., 2021).

A third example, which can be regarded as late stage as well, is the identification of subpopulations using one modality, which are then extracted and further analyzed using the second modality (Figure 2). This allows for cell type or state specific analysis of the second modality. As an example, cell types can be determined using a transcriptomic assay (e.g., scRNA-seq), followed by cell type-specific analysis of surface protein abundance (e.g., using CITE-seq) or regulatory landscapes (e.g., using scATAC-seq).

Finally, methods are either designed to handle specific combinations of modalities (see next paragraphs for examples), or for any combination of modalities. Examples of the latter that have been used extensively include the Weighted Nearest Neighbor (WNN) approach implemented in the widely used Seurat package (Hao et al., 2021), MOFA + which constructs a hybrid modality from paired modalities (Argelaguet et al., 2020), and LIGER which constructs a hybrid modality from unpaired data (Welch et al., 2019). While many challenges exist to improve multi-omic data integration methods, one key challenge is the inherent correction for differences between the modalities, including batch effects. More so than batch correction in unimodal single cell datasets it is a challenge to distinguish technical noise from biological variability. Although not many benchmarking studies are available, a recent study showed that WNN is particularly successful at integrating scRNA-seq and snATAC-seq data even in the presence of complex batch effects (Lee et al., 2023).

### 2.2 Transcriptome and surface proteome

The availability of commercial kits and protocols has increased the accessibility of combined profiling of the transcriptome and surface proteome. Computational approaches for the integration of paired transcriptome and surface proteome data have been increasingly needed for analyzing these datasets.

An example of a late-stage integration approach developed specifically for transcriptomic data and the surface proteome is CiteFuse (Kim et al., 2020). Cell-to-cell similarity matrices for both modalities are fused, which can then be used for clustering and dimensionality reduction. Results demonstrate that the integrated data enhances cell typing and other downstream analyses relative to single modality data (Kim et al., 2020). In contrast to CiteFuse, TotalVI (Gayoso et al., 2021) and sciPENN (Lakkis et al., 2022) are early-stage integration approaches and are based on neural network machine learning. Although these approaches were designed for integration of paired data, they do allow different cells to have different antibody panels in the protein modality. The neural network is used to impute the missing protein data, in effect relaxing the requirement of the data to be paired. This feature allows for adjusting the antibody panel in the lifetime of a project, while retaining the ability to integrate data obtained from different stages of the project.

### 2.3 Transcriptome and chromatin accessibility

Integration of transcriptomic and ATAC data is a special case as it can take advantage of ‘feature correspondence’ (Rautenstrauch et al., 2022). The scATAC peaks can be related to specific genes, and thus merged with the transcriptomic data. The result is a hybrid modality that can be used for downstream analysis, but unlike most other hybrid modalities, retains the ability for interpretation in the context of the genes. Many algorithms have been developed specifically for integration of transcriptomic data with scATAC-seq (see (Rautenstrauch et al., 2022) for an overview of the approaches). These various methods employ early- and late-stage frameworks and operate on both paired and unpaired data.

The combination of scRNA-seq and scATAC-seq modalities has proven useful in various ways, as illustrated in a recent study on mouse kidneys (Miao et al., 2021a). First, the combined modalities were able to define more distinct cell types compared to scRNA-seq analysis. Second, as an example of late-stage integration of scRNA-seq and scATAC modalities, the scRNA-seq and/or scATAC-seq modality can be used to annotate the cells, allowing identification of cell-type specific regulatory elements. Gene regulatory networks can then be inferred by relating active genes to active regulatory elements in each cell type (Cao et al., 2018).

### 2.4 Outlook on multi-omic integration methods

With the advent of single-cell methods came efforts to build cell atlases for tissues or even entire species (Regev et al., 2017). More recently, large datasets or reference maps of multi-modal data are also becoming available (Network, 2021). This introduced the need for multi-modal mapping of query datasets to reference maps. Several approaches have been developed specifically for this purpose (Lotfollahi et al., 2022a; Lotfollahi et al., 2022b), while other recent, more general methods are able to map very large datasets that involve different modalities (Hao et al., 2022). Development of such approaches will further expand the use of multi-omic single cell sequencing.

Community efforts such as the Multimodal single cell data integration challenge (Lance et al., 2022) can play important roles in guiding further method development. Notably, the competition winner, as well as recently developed integration approaches such as bridge learning (Hao et al., 2022), both lessen the distinction of paired and unpaired dataset: Small paired datasets are used to guide integration of the unpaired datasets, with an overall improved performance.

## 3 Overview of single cell studies in kidney diseases

Advances in single-cell sequencing technologies have enabled the analysis of individual cells obtained from tissues, such as kidney biopsies, and biofluids, such as urine and blood, at an unprecedented resolution, revealing cellular signatures of inflammation, cellular injury, and fibrosis in various kidney diseases. These signatures enable a deeper understanding of pathophysiology and can also facilitate development of precision therapeutics for these diseases. Single-cell profiling of kidney biopsies, PBMCs, urine samples and skin lesions from patients with lupus nephritis, acute kidney injury, diabetic nephropathy and focal segmental glomerulosclerosis present potential novel approaches for the diagnosis and monitoring of disease activity (Table 1). These approaches, when performed on urinary cells and PBMCs, in contrast to kidney biopsy, are non-invasive and could be repeated multiple times as needed. Several examples of studies performed using human samples are discussed below.

**TABLE 1:** Examples of multi-omic single cell sequencing studies in kidney diseases.

Condition	Technique	Source of samples	Key findings	Reference
**Lupus Nephritis**	scRNA-seq and urine proteomics	Kidney biopsy & Urine	• Identified 237 urinary biomarkers associated with lupus nephritis as compared to controls. Amongst these biomarkers IL-16, CD163, and TGF-β mirrored intrarenal nephritis activity	Fava et al. (2022)
			• IL-16 was found to be highly expressed at key sites of kidney injury and highlight its role in LN pathogenesis and a potentially treatable target and biomarker	
	scRNA-seq	Kidney biopsy & Urine	• The analysis identified multiple subsets of leukocytes including myeloid cells, T cells, natural killer cells and B cells that demonstrated both pro and anti-inflammatory properties, such as CD16^+^ macrophages, CD4^+^ TFH cells and FoxP3+Helios + regulatory T cells	Arazi et al. (2019)
			• Two chemokine receptors, CXCR4 and CX3CR1, were broadly expressed, suggesting a key role in cell trafficking	
			• Interferon response was observed in dividing CTLs and NK cells	
	scRNA-seq	Kidney & Skin biopsy	• Type I IFN-response genes in tubular cells and keratinocytes were much more highly expressed than those of healthy controls and was higher in non-responders, compared to treatment responders	Der et al. (2019)
			• Patients non-responsive to treatment demonstrate higher expression of fibrotic ECM proteins compared with responders	
**FSGS**	scRNA-seq	Urine	• Shed podocytes in the urine had loss of canonical podocyte markers, which are required for normal podocyte function, such as NPHS1, NPHS2, and PODXL.	Latt et al. (2022)
			• Patients with FSGS had higher expression of genes for epithelial-to-mesenchymal transition (EMT) compared to patients with MCD. These markers were relatively higher in treatment non-responders compared to responders	
	scRNA-seq	Kidney biopsy	• Highest glomerular endothelial cells (GEC) scores were observed in patients with FSGS	Menon et al. (2020)
			• Molecular endothelial signatures suggested 2 distinct FSGS patient subgroups with *α*-2 macroglobulin (A2M) as a key downstream mediator of the endothelial cell phenotype and was shown to have prognostic significance	
**AKI**	scRNA-seq	Urine	• Patients with pre-renal AKI excreted mainly myeloid cells, whereas urine samples from patients with AKI had higher expression of immune and epithelial cells	Klocke et al. (2022)
			• Tubular epithelial cells from urine samples of patients with AKI did not express characteristic segment markers, and instead showed injury-related dedifferentiation and adaptive phenotypes	
	scRNAseq, snRNAseq and spatial transcriptomics	Kidney biopsy	• This study localized the transcriptomic signature of various immune cells to spatial transcriptomic spots of known renal epithelial cells in ischemia reperfusion injury and cecal ligation puncture models of AKI	Melo Ferreira et al. (2021)
			• A subpopulation of injured proximal tubule cells with Activating Transcription Factor 3 (ATF3) and Midkine (Mdk) expression were identified, which may be responsible for neutrophil chemotaxis to the site of injury	
**Fibrosis**	scRNA-seq, ATAC-seq and spatial transcriptomics	Human & mouse kidney biopsy	• This study enabled mapping of all matrix-producing cells at high resolution, revealing distinct subpopulations of pericytes and fibroblasts as the major cellular sources of scar forming myofibroblasts during fibroblasts	Kuppe et al. (2021)
			• Using these data, myofibroblast-expressed Nkd2 was identified as a potential therapeutic target	
**Diabetic kidney Disease**	snRNA-seq	Kidney biopsy	• snRNAseq performed on cryopreserved human diabetic kidney samples.	Wilson et al. (2019)
			• It was seen that diabetic thick ascending limb, late distal convoluted tubule, and principal cells adopted a gene expression signature consistent with increased potassium secretion, including alterations in Na^+^/K^+^-ATPase, WNK1, mineralocorticoid receptor, and NEDD4L expression, along with increases angiogenic signaling	
	scRNA-seq and spatial transcriptomics	Kidney biopsy	• There was enrichment of specific cell subpopulations consisting of venous endothelial cells and fibroblasts with elevated expression of CCL21 and IGFBP5.	Chen et al. (2022)
			• Spatial analysis revealed that most of the immune cells were localized in areas of renal fibrosis	
	snRNA-seq & snATAC-seq	Kidney biopsy	• snRNA-seq and snATACseq performed on human DKD and non-diabetic kidney biosy samples and DKD was associated with an increased proportion of VCAM1+ proximal tubule cells (PT_VCAM1) and infiltrating leukocytes compared to non-diabetics	Wilson et al. (2022)
			• PT_VCAM1 cell is pro-inflammatory phenotype characterized by enhanced NFkB signaling and failed repair that may underlie transition from acute kidney injury to CKD	

### 3.1 Lupus nephritis (LN)

LN is a form of glomerulonephritis that constitutes one of the most severe organ manifestations of systemic lupus erythematosus (SLE). Despite increased knowledge of disease pathogenesis and improved treatment options, LN remains a substantial cause of morbidity and death among patients with SLE. Conventional markers of disease activity and response to therapy typically consist of measuring auto-antibody levels, markers of complement activity and laboratory parameters like proteinuria and estimated glomerular filtration rate (eGFR).

Single cell transcriptomics and urine proteomics were used to identify biomarkers that are upregulated in LN, including IL-16, which is found to be highly expressed at sites of kidney injury (Fava et al., 2022). Other studies using scRNA-seq on kidney biopsy and urinary cells have found evidence of upregulation of IFN response genes, fibrotic ECM proteins and chemokine receptors such as CXCR4 and CX3CR1 (Arazi et al., 2019; Der et al., 2019). These findings help our understanding of the pathogenesis of a complex disease like LN and may further help in predicting patients who may better respond to specific therapies.

### 3.2 Acute kidney injury (AKI)

AKI is a major health issue, the outcome of which depends primarily on damage and reparative processes of tubular epithelial cells. According to a recent meta analysis, 1 in 5 adults (21.6%) and 1 in 3 children (33.7%) experienced AKI worldwide (Susantitaphong et al., 2013). Mechanisms underlying AKI remain incompletely understood, effective therapies are lacking and monitoring the course of AKI in clinical routine is limited to measuring urine output and plasma levels of filtration markers. Hence, high-resolution approaches are needed to facilitate a better understanding of the pathogenesis of AKI and to potentially identify therapeutic targets in preventing and treating AKI.

In a recent study (Klocke et al., 2022), scRNA-seq analysis revealed that urinary cells from patients with established AKI had different transcriptional profiles compared to patients with pre-renal AKI, including markers of cellular dedifferentiation. Although single cell sequencing studies have improved our understanding of the transcriptomic signature of different cell types within the kidney, the spatial distribution of injury can be limited to certain regions in the kidney. In another study (Melo Ferreira et al., 2021), investigators were able to localize the transcriptomic signature of various immune cells to spatial transcriptomic spots of known renal epithelial cells in murine models of AKI. The analysis was able to detect a subpopulation of injured proximal tubule cells with Activating Transcription Factor 3 (ATF3) expression which may be responsible for neutrophil chemotaxis to the site of injury.

### 3.3 Focal segmental glomerulosclerosis (FSGS)

FSGS and minimal change disease (MCD) are common causes of nephrotic syndrome and share many common features, such as diffusely effaced podocytes. However, the response to treatment is variable in FSGS, and there is a higher risk of progression to chronic kidney disease (CKD) in patients with FSGS, compared to patients with MCD, who typically have a benign course. Multiple single cell studies (Menon et al., 2020; Latt et al., 2022) have identified specific markers such as *α*-2 macroglobulin (A2M), elevated glomerular endothelial cell score and loss of canonical podocyte markers in patients with FSGS compared to patients with MCD. These findings may help in differentiating FSGS from MCD and in predicting response to treatment (Menon et al., 2020).

### 3.4 Deconvoluation of kidney fibrosis

Fibrosis is a characteristic feature in all forms of CKD. Deposition of pathological matrix in the interstitial space and within the walls of glomerular capillaries as well as the cellular processes resulting in this deposition are increasingly recognized as principal factors resulting in progressive kidney damage. It has been challenging to study kidney fibrosis in patients, since kidney biopsies are usually not performed in patients with established CKD. Use of multi-omic techniques (Kuppe et al., 2021) have helped in identifying a population of scar-forming myofibroblasts. These techniques may help in identifying potential therapeutic targets for preventing fibrosis, such as the myofibroblasts identified in the aforementioned study.

### 3.5 Diabetic kidney disease (DKD)

DKD has a high global disease burden and substantially increases the risk of kidney failure and cardiovascular events. Despite treatment, there is substantial residual risk of disease progression with existing therapies. There is an urgent need to better understand the molecular mechanisms driving DKD to help identify new therapies that slow progression and reduce associated risks (Tuttle et al., 2022). snRNA-seq analysis of cryopreserved diabetic kidney samples showed upregulation of angiogenic and mineralocorticoid markers, consistent with the clinical manifestation of fluid overload and neo angiogenesis seen in patients with DKD (Wilson et al., 2019). Multi-omic techniques incorporating scRNA-seq and spatial transcriptome analyses have been used to generate an atlas of diabetic kidney disease. For example, in one study the investigators identified enrichment of specific cell subpopulations consisting of venous endothelial cells and fibroblasts with elevated expression of CCL21 and IGFBP5 (Chen et al., 2022). Furthermore, spatial analysis revealed that most of the immune cells were localized in areas of renal fibrosis. In another recent study, the investigators performed snRNA-seq, snATACseq and spatial transcriptomics on human DKD kidney biopsy samples and were able to identify increased proportion of VCAM1+ proximal tubule cells (PT_VCAM1) and infiltrating leukocytes compared to non-diabetics. These changes have adverse implications as they increase the pro-inflammatory milieu and facilitate AKI to CKD transition (Wilson et al., 2022). Thus, single cell sequencing technologies are helping to elucidate the underlying mechanisms of DKD.

## 4 Therapeutic development for kidney disease in single cell era

### 4.1 Target discovery: Genetic variants and gene perturbation

Single cell sequencing provides rich and complex phenotypic portraits of the cellular and molecular circuits involved in disease. These large datasets lead to the identification of an array of cellular and molecular features which are upregulated, downregulated or, in some cases, unique to disease states. Differences observed in disease vs. control samples provide hypotheses of new therapeutic targets, which may have become dysregulated at the cellular or molecular level. Nevertheless, challenges remain in deciphering which observed changes drive pathogenic processes versus those that are passenger effects.

Genetic variants and their loci from genome-wide association studies (GWAS) provide complementary evidence to single cell sequencing datasets for the involvement of genes in the development of diseases. A challenge, however, in identifying specific targets from genomic variant studies is that the large majority of disease-associated loci are in non-coding regions, thus complicating their linkage to specific gene products or cell types that could be candidates for therapies. By integrating single cell sequencing datasets with GWAS candidate genes/loci, greater linkages can be established to identify genes, pathways or cell types with causal relationships to disease. In one analytical framework, genes near disease-associated loci from GWAS studies are compared to various cell subsets identified by scRNA-seq to nominate disease-relevant cell subtypes and pathways. This framework led to the identification of cell clusters enriched for expression of TLR7 (nucleic acid sensing), HIP1 (endocytic participating protein implicated in DC regulation), and LBH (modulates synovial hyperplasia) in LN patients (Arazi et al., 2019). When multiple genes in a shared pathway are implicated for a disease, a gene signature incorporating a panel of genes, rather than an individual gene, can be used to identify potential disease-relevant cell types and pathways in single cell data. For example, multiple loci related to the type I interferon pathway have been implicated in SLE disease activity by GWAS studies (Rice et al., 2017; Psarras et al., 2022) and gene signature modules of interferon-stimulated genes (ISG) have been shown to correlate with SLE disease activity. When these modules were assessed for enrichment in cell clusters identified in pediatric SLE patients by scRNA-seq, multiple distinct subpopulations were identified (Nehar-Belaid et al., 2020).

Since functionally important gene regulatory regions are mostly nucleosome-free, chromatin accessibility data can be used to aid understanding of candidate risk loci in non-coding regions. Additionally, expressed quantitative loci (eQTL) approaches are commonly used to map variants to causal genes. However, a critical limitation in chromatin accessibility and eQTL analyses for mapping causal genes arises from cell type heterogeneity; bulk methods capture aggregated expression or accessibility across multiple cell types, with cell type diversity and proportionality complicating interpretation [reviewed in (Maria et al., 2022)]. In a pioneering study for kidney disease, Sheng, et al. used single cell multi-omics (scRNA-seq and snATAC-seq), human genetic information, and advanced computational approaches to demonstrate how genetic variants render a functional effect on cell types and specific gene/pathway programs, resulting in the identification of more than 200 genes involved in kidney function and hypertension (Sheng et al., 2021). More recent studies have expanded on this work, providing further mapping of risk alleles to specific cell types, pathways, and genes (Liu et al., 2022; Sandholm et al., 2022), which can augment identification of new targets (Doke et al., 2021a; Doke et al., 2021b). In another study, whole kidney and single cell epigenomic information of hundreds of samples was used with GWAS data to define the genetic association of kidney function in 1.5 million individuals, resulting in identification of 878 loci (126 novel), with prioritized target genes for 87% of the loci (Liu et al., 2022). Collectively, the results have provided meaningful insights to pathologic cell types and disease-causing pathways, which can guide identification of new therapeutic targets.

To further facilitate target identification and drug discovery, single cell profiling has been combined with gene perturbation methods as a forward genetics screening approach to explore phenotypic impacts of gene modulation. Perturb-seq and related approaches integrate pooled CRISPR screening with single cell profiling, enabling systematic determination of the impact of inhibitory and activating perturbations to large numbers of candidate genes (Adamson et al., 2016; Dixit et al., 2016; Jaitin et al., 2016). The phenotypic readouts can identify cell states that are desirable for therapeutic intervention, and can be applied in more complex biological systems, including co-cultures, organoids and in animal models. Perturb-seq methods have been extended to understand the role of non-coding genetic variants associated with disease (Gasperini et al., 2019) and, in combination with CITE-seq, define mechanisms of cancer immune evasion (Frangieh et al., 2021). These forward genetic methods provide complementary information for a deeper phenotypic understanding of the impact of candidate genes as potential therapeutic targets.

### 4.2 High-resolution diagnostics and biomarkers in kidney disease

A high-resolution picture of all cell types from a disease tissue sample provides a comprehensive and deep interpretation of the underlying biology and should be informative to diagnostics and prognostics. Single cell sequencing can provide a comprehensive capturing of all cell types, as well as information regarding their molecular pathways, thereby providing greater detail of disease endotypes and potential for therapy responsiveness. Indeed, studies have used single-cell profiling of peripheral blood immune cells to identify correlates of anti-PD1 responsiveness in cancer patients (Wu et al., 2020; Luoma et al., 2022).

For diagnostics and biomarkers, a high-resolution molecular picture of the kidney would be of great value, however, a key challenge in molecular diagnostics, including single cell profiling, for kidney disease is the limited ability to obtain kidney biopsy samples for such analyses. As performing a kidney biopsy carries some risk of complications for patients, they are commonly performed at time of diagnosis and at limited additional timepoints, as needed for patient care. Core biopsies contain a relatively low number of cells (single digit thousands for single cell profiling). Moreover, the tissue must be dissociated to single cell suspension, which can introduce artifacts, such as protease-induced changes in gene expression, and lead to biased loss of cell types. To address these key challenges in diagnostics and biomarkers, emerging studies have begun to analyze surrogate tissue sources of cells from urine for single cell profiling, as well as spatial profiling from limited tissue derived from kidney biopsy cores.

Urine sedimentary analysis has been used for decades to inform diagnoses for kidney diseases, however, until the development of single cell profiling approaches, robust and accessible methods to generate an unbiased determination of cell types and their molecular features were lacking. Single cell profiling of urinary cells from DKD patients demonstrated an ability to detect nearly all cell types of the kidney (Abedini et al., 2021). Emerging studies applying single cell sequencing to urinary cells in FSGS (Latt et al., 2022), lupus nephritis (Arazi et al., 2019), and AKI (Cheung et al., 2022; Klocke et al., 2022) have underscored the potential of using this non-invasive cell source for diagnostic and other disease insights. TCR analyses of patients with immune checkpoint-associated nephritis revealed the T cell clonotypes in the kidney are enriched in urine, supporting a direct linkage between T cells in urine and kidney (Singh et al., 2022). In a recent study of acute cell rejection (ACR) of kidney transplantation, investigators applied single cell RNA and TCR sequencing to biopsies from allografts as well as urinary cells (Shi et al., 2023). Interestingly, TCR sequences associated with the expanded CD8 T cell population were also observed in matching urine samples. These results underscore a linkage between immune cellular phenotypes from urine and kidney tissue, and relate this information to treatment response. Collectively, while initial studies showcase the opportunity to be harnessed by applying single cell sequencing to urinary cells, additional research is needed to better define the relationships between urinary cells and disease biology and progression.

Spatially resolved omic profiling methods, largely rooted in transcriptomics, address multiple challenges with the limited tissue from kidney biopsies. These methods do not require dissociation of tissue, thereby retaining the spatial biological context of cells and eliminating sample biases and loss of cells from dissociation methods. Furthermore, spatial profiling methods often require limited tissue amounts and can be compatible with formalin-fixed, paraffin-embedded (FFPE) tissues, making them more amenable to the limited material of kidney core biopsies from clinical settings that use FFPE as standard of practice. Commercialization of spatial transcriptomic methods has reduced the technical barriers of access to these complex methods, with FFPE-compatible platforms including from 10x Genomics (Visium and Xenium), Nanostring (GeoMx, nCounter, CosMx), Vizgen (MERSCOPE), among others. The technologies are rooted in one of two fundamental methods: *in situ* hybridization (ISH) and next-generation sequencing (NGS)-based methods. ISH methods can provide subcellular localization information but are limited to a selected (and therefore biased) set of several hundred gene probes. In contrast, NGS-based spatial transcriptomics methods combine scRNA-seq technology with spatial barcodes on a specialized slide, enabling amplification of copied transcripts while retaining localization information. The NGS-method provides unbiased, genome-wide transcriptomics but is limited in spatial resolution due to the spot size on the slide. The current 10x Visium methodology incorporates spot sizes of 55 μm, which falls short of single-cell resolution. To improve the resolution, separate conventional (non-spatial) scRNA-seq data can be integrated with the spatial data, and combined with computational deconvolution methods, used to estimate single cell contributions (see reviews (Melo Ferreira et al., 2021; Rao et al., 2021; Zhang et al., 2023). Studies incorporating spatial transcriptomic profiling methods on human kidney samples are emerging, with examples including AKI to understand immune cell infiltration in histological context (Melo Ferreira et al., 2021), development of an atlas across multiple kidney diseases (Lake et al., 2021), cell-mediated rejection in kidney transplantation (Salem et al., 2022), and small RNA involvement in FSGS (Williams et al., 2022). Multi-omic single cell approaches incorporating spatial profiling have been reported, for example, to understand fibrosis microenvironments in diabetic and hypertensive diseased human kidneys (Abedini et al., 2022). Spatial profiling methods are a powerful emerging technology compatible with kidney biopsy samples that will bring greater understanding of disease processes and treatments.

## 5 Summary and outlook

Multi-omic single cell sequencing has facilitated the creation of high-resolution cellular and molecular maps in the context of kidney disease, providing new insights into disease mechanisms and opportunities for therapeutic intervention. New experimental methods enable measurement of multiple molecular features simultaneously, including gene expression, surface protein expression, TCR/BCR sequences and chromatin accessibility. Computational tools to integrate these large and diverse datasets have enabled derivation of rich biological insights, from pathogenic mechanisms to new therapeutic targets. And while most studies applying single cell sequencing methods in the context of kidney diseases have utilized single modality datasets (i.e., gene expression), increasingly multi-omic approaches are being pursued.

Single cell profiling is increasingly accessible to researchers, due to developments of more user-friendly experimental workflows (e.g., with commercial kits) and data analysis tools (e.g., graphical interface software). However, tissue availability from kidney core biopsies has been a major limitation for single cell profiling in the context of kidney disease. Emerging alternative approaches compatible with single cell profiling are being developed, including urinary cell analysis and spatial profiling. With studies in which nearly all kidney cell types can be identified in urine by single cell profiling, the potential for routine, non-invasive monitoring of biological changes of individual diseased patients is an exciting one, with ramifications for more optimal treatment selections (e.g., therapeutic classes and doses). These developments portend a future of collection of these high-resolution datasets in a variety of clinical settings, from observational studies to therapeutic interventional trials. Collectively, these advancements are heralding new opportunities for precision medicine in kidney disease, from diagnostics and patient segmentation to prognostics and new targeted therapies, with the potential to better match the right therapies with the right patients at the right time.
